# Effects of Pulsatile and Non-Pulsatile Cardiopulmonary Bypass Techniques in Coronary Artery Bypass Grafting Surgeries on Cerebral Perfusion

**DOI:** 10.4274/TJAR.2024.231331

**Published:** 2024-02-28

**Authors:** İpek Bostancı, Beyhan Güner, Evrim Kucur Tülübaş, Güray Demir, Zafer Çukurova

**Affiliations:** 1 University of Health Sciences Turkey, Bakırköy Dr. Sadi Konuk Training and Research Hospital, Clinic of Anaesthesiology and Reanimation, İstanbul, Turkey; 2 University of Health Sciences Turkey, Taksim Training and Research Hospital, Clinic of Anaesthesiology and Reanimation, İstanbul, Turkey

**Keywords:** Cardiopulmonary bypass, cardiovascular and thoracic anaesthesia, near-infrared spectroscopy, postoperative cognitive disfunction, pulsatile flow, S100β protein

## Abstract

**Objective:**

We aimed to evaluate the effects of cardiopulmonary bypass (CPB) machines used in coronary artery bypass grafting surgeries on cerebral perfusion by performing cerebral oximetry monitoring [near-infrared spectroscopy (NIRS)], S100-β protein measurements, and neurocognitive function assessment tests using both pulsatile and non-pulsatile modes.

**Methods:**

A total of 44 patients, 22 non-pulsatile (Group NP) and 22 pulsatile (Group P), were included in the study. Hemodynamic parameters, arterial blood gas values, NIRS values and blood S100β protein levels were analyzed at five points: pre-induction (T1), initiation of CPB (T2), termination of CPB (T3), end of surgery (T4), and postoperative 24 h (T5). Two different neuropsychological tests were administered to patients in the preoperative and postoperative periods.

**Results:**

There were no significant differences between the groups for demographic characteristics such as age, gender, body mass index, aortic cross-clamping, CPB, and operation durations. The mean arterial blood pressure and PaO_2_ values for the T2 measurements were significantly higher in group NP (*P* < 0.05). Regional cerebral oxygen saturation (rSO_2_) (NIRS) values at T3 and T4 were significantly higher in group P (*P* < 0.05). Serum S100β measurement values at T3 and T5 were significantly higher in group NP than in group P (*P* < 0.05). Serum S100β protein levels at T3 correlate with rSO_2_ results. There was no statistically significant difference between the two groups in terms of pH, lactate, glucose, partial pressure of carbon dioxide, and peripheral oxygen saturation values.

**Conclusion:**

Despite no difference between the two groups for neurocognitive function tests, we believe that pulsatile perfusion may be more beneficial for cerebral perfusion when S100β protein and NIRS values are considered. Further clinical studies are needed to evaluate the benefits of the pulsatile technique for cerebral perfusion.

**Main Points**

• The effects of the pulsatile technique and non-pulsatile technique using the cardiopulmonary bypass (CPB) machine were compared for postoperative cerebral functions. Pulsatile pump flow was found to be more beneficial according to regional cerebral oxygen saturation (rSO_2_) and S100β protein results.

• The rSO_2_ recorded in the T3 and T4 periods were significantly higher in group P than in group NP, representing better cerebral perfusion with pulsatile CPB flow.

• S100β values were lower at T3 and T5 time intervals with pulsatile perfusion.

• According to the results of neurocognitive tests, no significant difference was found between the groups in terms of postoperative cognitive dysfunction.

## Introduction

In the mid-20^th^ century, the cardiopulmonary bypass (CPB) machine developed by John Gibbon enabled surgery to be performed for many cardiac malformations, such as coronary artery disease, valve repair, and replacement. However, it also has some negative effects on the organs it perfuses. The brain, which is highly sensitive to hypoxia, may be affected by thromboembolic ischemic events, bleeding, or inflammatory responses secondary to the procedures that occur during CPB.^1^

After cardiac surgery, major neurological complications (intracranial hemorrhage, ischemic stroke, etc.), neurocognitive dysfunction, and subclinical neurological deficits can be observed. In addition, cognitive function disorders such as memory loss, concentration impairment, and loss of fine motor skills that develop after the use of CPB have also been reported. However, there is still no consensus regarding the incidence, etiological causes, diagnostic methods, and course of these complications.^2^

Imaging methods involve some clinical difficulties with their use for diagnostic purposes after the development of complications and lack of bedside use. Near-infrared spectroscopy (NIRS) is a technology that interprets oxy- and deoxyhemoglobin signals to measure regional cerebral oxygen saturation (rSO_2_) in real time. NIRS is a non-invasive, easy-to-use, and inexpensive method that has proven feasibility and safety during cardiac surgery. It has all the theoretical advantages that make it the gold standard for real-time cerebral monitoring in cardiac surgery, as it measures cerebral oxygenation independently of brain function, metabolism, and cerebral blood flow.^3^

No universally accepted definition of postoperative cognitive decline, or dysfunction (POCD) has been developed, and its pathogenesis remains unknown. The statistical criteria and methods used to define POCD, the selection and evaluation of neuropsychological tests, and the evaluation time of assessment are primarily left to the discretion of the authors, resulting in a wide variation of POCD incidence in different studies.^4,5^

Currently, there is interest in using biochemical markers because of the insufficient sensitivity of neuropsychological tests for the diagnosis of neurological disorders^6^ that develop after cardiac surgery. S100β and neuron-specific enolase (NSE) proteins, which are specific proteins originating from neurons, can be used as neurobiochemical markers because they are linked to stroke, traumatic brain injury, cardiac arrest, and brain damage after CPB.^7^

Our aim was to investigate the effects of non-pulsatile and pulsatile CPB pumps on cerebral circulation by measuring S100β protein and monitoring NIRS. In addition to these measurements, we aimed to observe whether there was a deterioration in postoperative basic neurocognitive functions by comparing preoperative and postoperative mini-mental test scores.

## Methods

After obtaining approval from the University of Health Sciences Turkey, Bakırköy Dr. Sadi Konuk Training and Research Hospital, Clinical Research Ethics Committee with protocol code 2012/15/08 and date 05.11.2012, coronary artery bypass grafting surgery was planned under elective conditions. A total of 44 patients who were over the age of 18 and under the age of 70, with ejection fraction more than 40%, and who had adequate educational attainment to perform neurocognitive testing were included, while patients with severe cardiac insufficiency and carotid stenosis, a previous history of cardiovascular accident, and renal failure were excluded.

The patients were verbally and in writing informed, and written informed consent forms were obtained.

The patients were randomized into two groups. Group P (n=22) received pulsatile CPB, and Group NP (n=22) received non-pulsatile CPB.

Transient POCD and postoperative delirium (POD) are relatively common complications after surgery. Patients undergoing cardiac surgery are at high risk of both conditions, but the predisposing cognitive profile for these conditions has not yet been fully elucidated.^8^

According to The American Psychiatric Association’s fifth edition of the Diagnostic and Statistical Manual of Mental Disorders, delirium is defined as a condition with the following five key features: disturbance in attention and awareness; the disturbance develops over a short period of time and its severity tends to fluctuate during a day; an additional disturbance in cognition; these mentioned disturbances cannot be better explained by other pre-existing neurocognitive disorders and do not occur in severely reduced arousal level such as coma; and there is evidence suggesting the disturbance is a direct result of another medical condition.^9^

Mini-Mental State Examination (MMSE) is the most widely recognized and used brief screening instrument for detecting cognitive deficits. Both the MMSE and the Montreal Cognitive Assessment (MoCA) are brief cognitive screening tools that are administered in a paper-and-pencil format. For both tests, a score is derived by summing the points from each successfully completed task, for a total range of 0-30 points; higher scores indicate better cognitive performance. MoCA was developed to screen milder forms of cognitive impairment through the assessment of a wide range of cognitive functions, such as short-term memory, executive functions, visuospatial abilities, language, attention, concentration, working memory, and temporal and spatial orientation by Nasreddine et al.^11^ in 2005.^10^

The standardized MMSE (SMMSE) and the MoCA were administered preoperatively one day before surgery and postoperatively on the 7^th^ day to evaluate the neurocognitive functions of the patients. Reliability and validity studies for the Turkish form of MMSE were conducted by Güngen et al.^12^ in 2002.

At the beginning of the operation, all patients were monitored with electrocardiogram (ECG), non-invasive blood pressure, peripheral oxygen saturation (SPO_2_), NIRS, and bispectral index (BIS) for depth of anaesthesia. Subsequently, invasive blood pressure monitoring was performed. During induction, 0.1-0.2 mg kg^-1^ of midazolam, 5-8 µg kg^-1^ of fentanyl, and 0.6 mg kg^-1^ of rocuronium were administered to the patients. The aim was to maintain the depth of anaesthesia in the range of 40-60, which is the BIS general anaesthesia level for sevoflurane inhalation and remifentanil infusion.

The pump priming fluid was prepared with 1000 cc Isolate S, 100 cc 20% mannitol, and 100 mg heparin. CPB was initiated by aortoatrial cannulation in all cases. The CPB circuit included a roller pump (Stöckert SV, C5), 40 µm arterial filter, adult membrane oxygenator, and hard-shell venous cardiotomy reservoir. The pump flow was adjusted to set the mean arterial blood pressure (MAP) at 50-70 mmHg during CPB. The nasopharyngeal body temperature was lowered to 32 °C. Following the placement of an aortic cross-clamping (ACC), non-pulsatile perfusion was applied to the first group and pulsatile perfusion was applied to the second group. The pump module was used to run pulsatile pump flow control with an internal ECG simulator during total bypass. The flow characteristics were determined by selecting the pump usage percentage and continuous basal flow for each ECG cycle. The heart rate was set to 60-70 rpm in the pulsatile mode. The pulse width was adjusted to 40-50% and the basal flow amount was set to 35-50%.

The demographic characteristics of the patients [age, gender, and body mass index (BMI)], and the durations of the operation, ACC, and CPB were recorded. The levels of MAP, SPO_2_, right and left rSO_2_, partial pressures of oxygen and carbon dioxide (PaO_2_, PaCO_2_), pH, lactate, glucose and S100β protein were evaluated and recorded before the induction of anaesthesia (T1), at the beginning of CP, after 5 min of ACC (T2), at the termination of CPB after ACC remova, before weaning (T3), at the end of the operation (T4), and in the postoperative 24^th^ hour (T5) (Table 1).

Human S100β was tested using the Bio Vendor (Czech Republic) ELISA method. The standards, quality control serum, and patient samples were pipetted onto polyclonal antibody-coated microplates with the catalog number RD192090100R. After 120 min of incubation, the washing procedure and number of washes were performed as suggested in the kit protocol, followed by the addition of biotin-labeled monoclonal anti-human S100β antibody. A second incubation of 60 min was performed, and the washing procedure was repeated. After the conjugate and substrate pipetting procedure was completed, the reaction was stopped and a reading was obtained at 450 nm. Logarithmic graphs were drawn for absorbances and converted to pg mL^-1^ for concentration. The intra-assay coefficient of variation (CV) values for the kit were reported as 3.8%, and the inter-assay CV values were 5.2%.

Before the study, a preliminary study of 5 cases was conducted using Group NP and Group P. The mean and standard deviation values for S100β protein levels measured in the T5 period for Group NP and Group P were calculated as 60±50 pg mL^-1^ and 30±20 pg mL^-1^, respectively. In the power analysis made according to these values, the number of patients to be included in each group was calculated as 21, a total of 42, in order for the power of the study to reach 80% with a margin of error of α=0.05 (GPower 3.1).

### Statistical Analysis

The data homogeneity of the groups was evaluated using the Shapiro-Wilk test. Student’s t-test was used for paired group comparisons. For statistical representation, the mean and standard deviation were used. Values with *P* < 0.05 were considered statistically significant. The statistical analysis in this study used the SPSS V22 statistical program. As the primary outcome of the study, S100β levels measured in the postoperative 24^th^ hour (T5) were determined for both groups.

## Results

There were no significant differences between the groups for demographic characteristics such as age, gender, BMI, ACC, CPB, and operation durations (Table 1).

Before anaesthesia induction (T1), at CPB initiation (T2), end of CPB (T3), end of the operation (T4), and at postoperative 24^th^ hour (T5), there were no significant differences between MAP, SPO_2_, blood pH, pO_2_, partial pressure of carbon dioxide, lactate level, and glucose level (Table 2).

Comparison of rSO_2_ right and rSO_2_ left values showed that rSO_2_ right and rSO_2_ left values were significantly higher in Group P than in Group NP at termination of CPB (T3) and end of operation (T4) (*P* < 0.05). No statistically significant difference was found between the groups in terms of the averages for the other levels (*P* > 0.05) (Table 3).

When comparing the average levels of S100β protein between the groups, the levels of S100β protein in Group NP at termination of CPB (T3) and 24 h after the operation (T5) were significantly higher than those in Group P (*P* < 0.05). No statistically significant difference was found between the groups in terms of S100β protein levels recorded at other time intervals (*P* > 0.05) (Table 4).

No statistically significant differences were found between the pre- and post-operative averages for the SMMSE and MoCA between the groups (*P* > 0.05) (Table 5).

## Discussion

Cardiac surgery differs from other types of surgeries because of the potential for different complications related to the CPB machine. Contact of blood with synthetic surfaces in CPB equipment causes vasospasm, platelet-endothelial cell interactions, and increased inflammatory response, which may result in decreased microcirculation of the heart, brain, and other organs that can cause dysfunction of these organs.^13^ Current research targets the reduction of these negative effects and prevention of potential complications of CPB and its components, while discussions and studies about the perfusion method continue.^14^

In this study, we compared the effects of the pulsatile technique with the CPB machine on postoperative cerebral functions. Although significant differences were found for rSO_2_ and S100β protein results in the perioperative period with the use of the pulsatile perfusion technique, which is relevant to our hypothesis that pulsatile CPB flow may be more beneficial for cerebral perfusion, the two techniques were similar in terms of postoperative cognitive function. In contrast to the pulsatile flow present in normal circulation, CPB has been developed with a non-physiological laminar flow profile. This pulsatile flow is a necessary factor for sustaining adequate microcirculation and providing oxygen and nourishment to the internal organs.

However, because CPB is traditionally used with the non-pulsatile technique, this non-physiological blood flow may have adverse effects on microcirculatory perfusion. The pulsatile perfusion technique has not been adopted as a routine technique because the idea that pulsatile flow may provide extra benefits during CPB contradicts the idea that pulsatility can damage blood cells. In a systematic review of post-CPB microcirculatory disturbances, the microvascular flow index decreased during CPB, which may lead to a microcirculatory disorder.^15^ In this compilation, three studies compared pulsatile blood flow during CPB with non-pulsatile blood flow.^16,17,18^ Koning et al.^17,18^ reported that microcirculatory perfusion was protected following weaning from CPB with pulsatile blood flow compared with non-pulsatile flow using sidestream dark field imaging to evaluate sublingual mucosal microvascular perfusion, while O’Neil et al.’s^16^ study reported that microcirculatory perfusion was preserved with pulsatile flow compared with non-pulsatile flow during CPB. In a study by Zhao et al.^19^ of 40 infants with Fallot tetralogy, microcirculation improvement was described as an advantage of pulsatile flow; however, they also noted that pulsatile pumps could have the potential disadvantages of higher levels of hemolysis and potential platelet activation. Despite the extensive literature and increasing number of studies, the question of whether pulsatile perfusion flow is superior to non-pulsatile flow during CPB remains unanswered.^20,21^ Considering that pulsatile flow may be beneficial in cardiac surgery despite the different focuses and results in the literature, we aimed to observe its possible beneficial effects on cerebral circulation.

Many studies have summarized the benefits of NIRS in preventing potential catastrophic neurological events that cannot be detected using conventional monitoring. Cardiac surgery patients with significant decreases in rSO_2_ from baseline values are at increased risk of POCD, delirium, and longer intensive care unit and hospital stays.^22^

According to a study on POCD, patients with POCD had lower perioperative cerebral rSO_2_ than those without POCD. The decrease in rSO_2_ played an important role in the development of POCD, offering an appropriate monitoring method and potential treatment target.^23^

Therefore, in this study, cerebral oxygen saturation was monitored using NIRS. The rSO_2_ values recorded at T3 and T4 periods were found to be significantly higher in group P, representing better cerebral perfusion with pulsatile CPB flow, whereas the neuropsychological test results of our study did not correlate with cerebral rSO_2_ values.

Due to advances in medical techniques, the rates of major complications (e.g., mortality) following cardiac surgery have decreased, whereas the incidence of POCD has remained unchanged and it has become the most common postoperative complication.^24^ A recently formed multinational, multidisciplinary, and multispeciality expert group (Perioperative Cognition Nomenclature Consensus Working Group) recommended that cognitive impairment identified in the perioperative period be called “perioperative neurocognitive disorders” (PND). In addition, no universal neuropsychological testing method has yet been proposed by the group, and cognitive domains (such as memory, attention, visual-spatial organization, verbal fluency, motor function, and processing speed) considered for testing have not been defined.^25,26^ Currently, multiple tests are advocated for the diagnosis of PND. Therefore, in the present study, the SMMSE, which is the most widely recognized and used brief screening instrument for detecting cognitive deficits, and the MoCA, which is a brief instrument developed for the screening of milder forms of cognitive impairment, having surpassed the well-known limitations of the MMSE,^10^ were applied to patients one day before the operation and on the seventh postoperative day to identify cognitive disorders that may occur after cardiac surgery. The incidence of POCD varies widely in different studies because the statistical criteria and methods used to define POCD, neuropsychological test selection, and evaluation duration are left to the discretion of clinicians.^27^ According to the results of the neurocognitive tests conducted in our study, no significant difference was found between the groups in terms of POCD.

Despite the efforts made and the progress in surgical techniques during the past decades, the incidence of delirium after cardiac surgery remains between 26% and 52% when estimated with rigorous methodology.^28^ POD and POCD are common complications of cardiac surgery. It is unknown whether they have a similar etiology and pathophysiology. The relationship between POD and POCD is complex and not yet fully elucidated. Both entities share many risk factors, such as increasing age, low level of education, and underlying comorbidities, and might be viewed as two expressions of the same underlying process of pre-existing decreased cognitive reserve, as opposed to other evidence supporting a more independent, possibly causal relationship between delirium and cognitive impairment. A causal relationship could have important clinical implications because delirium would then be one of the few modifiable risk factors for POCD, opening up possibilities for prevention. The magnitude of the influence of delirium as an independent risk factor for POCD is difficult to determine.^8^

S100β protein can be found in glial and Schwann cells but cannot be detected in serum except in patients with significant medical pathologies. According to an article that investigated neurocognitive function and biochemical markers after cardiac surgery, medical conditions such as paralysis, subarachnoid hemorrhage, head trauma, CPB, and coma after cardiac arrest can lead to an increase in serum S100β protein.^29^

In an article investigating delirium after cardiac surgery, the ideal delirium indicator should have high sensitivity and specificity, be associated with the severity of the disease, be stable, easily accessible, independent of physiological variables, cost efficient, easily identifiable, and have high validity, as well as be associated with a known mechanism such as localized damage.^30^ However, developing an ideal marker for delirium or POCD is a complex process because there are many intricate factors that contribute to the occurrence of PND.

According to a study on cognitive dysfunction after CABG surgery, additional evidence suggested that CABG surgery with CPB was associated with high postoperative serum S100β protein and NSE levels, which can indicate significant neural damage, and that S100β protein serum levels may be more accurate than NSE in predicting POCD. They also reported that using a test panel instead of a single biomarker may provide more benefit for the early diagnosis of delirium after cardiac surgery.^7^

Fazio et al.^31^ showed that the elevation of S100β during and after cardiothoracic surgery is associated with perioperative factors such as the presence of extracorporeal pumps, use of cell savers, and degree of perfusion, in addition to patient-related factors such as age, gender, and the presence of hypertension. In the present study, the analysis of S100β protein used serum biomarkers and pulsatile perfusion flow was more advantageous based on S100β protein values at times T3 and T5 (Table 5), which may represent significant neural damage during non-pulsatile CPB flow. Despite many studies analyzing serum S100β levels to evaluate neurological dysfunction that may arise because of the surgical procedure in patients who have undergone cardiac surgery, the relationship between S100β levels and neurological and neurophysiological findings has not been fully defined.^29^

However, no biomarker has been found with sufficient sensitivity and specificity to be the gold standard determinant of neurological dysfunction after cardiac surgery. In clinical studies conducted in a similar manner, no single method was sufficient for the diagnosis of POCD. Therefore, in the present study, while investigating the cerebral effects of pulsatile perfusion during CPB, biochemical biomarkers, rSO_2_, and neuropsychological tests were applied, and the correlation between these methods was evaluated. As a result, rSO_2_ values were higher at T3 and T4 time intervals with pulsatile perfusion, while S100β values were lower at T3 and T5 time intervals, which may indicate better cerebral perfusion.

### Study Limitations

Our study has a few limitations. First, the lack of correlation between neurological monitoring and neuron-specific biomarker results and neurocognitive tests may have been due to the small number of cases. Second, the results may not reflect the general population because the study was conducted in a single center with a limited number of patients.

## Conclusion

In conclusion, while potential beneficial effects of pulsatile perfusion with the CPB machine on neurocognitive functions were observed, there is no gold standard test to diagnose potential neurologic disorders. We believe that follow-up, treatment, and diagnosis protocols should be established, including the perfusion technique, to identify and prevent any cerebral perfusion disorders that may occur during and after CPB. Therefore, we believe that larger-scale studies will be beneficial and provide results that are consistent with clinical data that may lead to routine use of pulsatile CPB flow.

## Figures and Tables

**Table 1:**
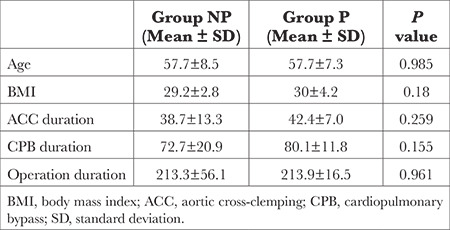
Comparison of Characteristics Between Groups

**Table 2:**
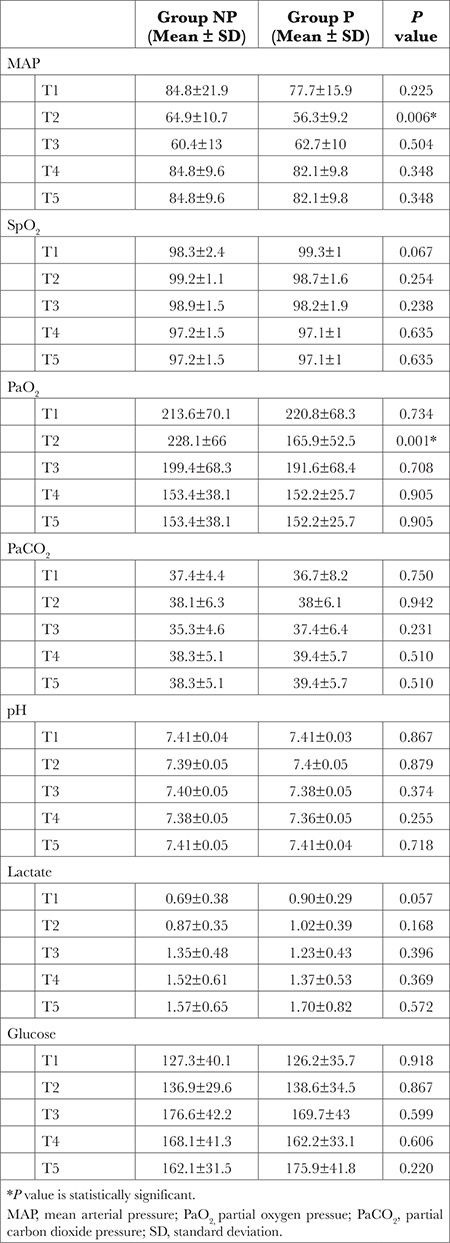
Comparison of Characteristics Between Groups

**Table 3:**
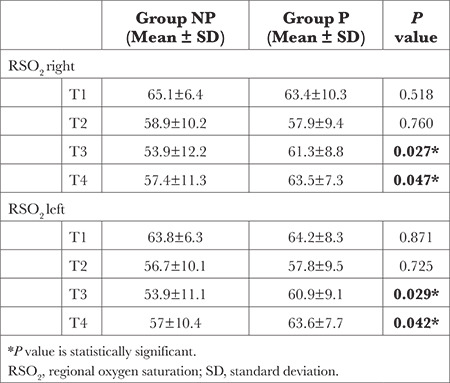
Comparison of rSO_2_ Right and rSO_2_ Left Values Between Groups

**Table 4:**
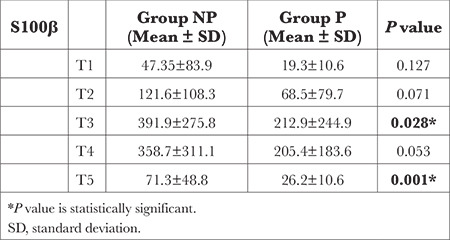
Comparison of S100β Protein Levels Between Groups

**Table 5:**
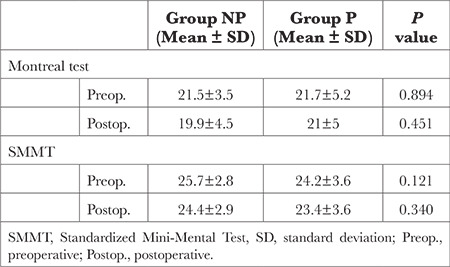
Comparison of Montreal Test and SMMT Between Groups
